# Optimization and validation of the HIV-1 neutralizing antibody assay in A3R5 cells

**DOI:** 10.1186/1742-4690-9-S2-P69

**Published:** 2012-09-13

**Authors:** M Sarzotti-Kelsoe, X Daniell, CA Todd, M Bilska, C LaBranche, LG Perez, C Ochsenbauer, J Kappes, W Rountree, DA Ozaki, JH Kim, R McLinden, T Denny, DC Montefiori

**Affiliations:** 1 Duke University Medical Center, Durham, NC, USA; 2 University of Alabama, Birmingham, AL, USA; 3 U.S. Military HIV Research Program, WRAIR, Silver Spring, MD, USA

## Background

A3R5 is a highly sensitive cell line for the detection of neutralizing antibodies (Nabs) against tier 2 strains of HIV-1. This cell line is particularly useful for the detection of weak Nab responses in preclinical and clinical trials of candidate HIV-1 vaccines. All methods used for endpoint analyses in clinical trials should be validated and demonstrably fit for purpose, in compliance with ICH Q2 (R1) guidelines. Here we describe the optimization/qualification and validation of the HIV-1 Nab Assay in A3R5 cells.

## Methods

A3R5 is a human lymphoblastoid cell line naturally expressing CD4 and CXCR4 and engineered to express CCR5. Nab assays in A3R5 cells are performed with Env.IMC.LucR viruses containing a reporter gene in the viral genome, whose expression is induced by viral Tat protein soon after infection. Luciferase activity is quantified by luminescence and is directly proportional to the number of infectious virus particles present in the viral inoculum. The assay is performed in 96-well culture plates for high throughput capacity.

## Results

We determined the stability of the cell line over time in culture for receptor and coreceptor expression, susceptibility to infection, and sensitivity to neutralization. The assay was optimized for cell density, input virus dose, length of incubation time and use of DEAE-dextran. We also determined the stability of a set of reference reagents, for validation experiments and for future competency and proficiency testing that express a broad spectrum of neutralization phenotypes. A prospective validation plan with pre-set pass/fail criteria was composed and implemented that addressed key assay parameters, including accuracy, precision, limit of detection and quantitation, specificity, linearity and range, robustness and specificity. Results of validation experiments were statistically analyzed and used to generate a final validation document.

## Conclusion

This validated assay will be used to identify correlates of protection in HIV vaccine trials conducted globally.

